# Predicting the Potential Applicability to Other Technical Fields Through the Linkage Between Backward and Forward Citations

**DOI:** 10.3389/frma.2021.736687

**Published:** 2021-10-14

**Authors:** Masayuki Hirose

**Affiliations:** Ph.D. Program, Graduate School of Business Administration, Hitotsubashi University, Tokyo, Japan

**Keywords:** innovation study, patent analysis, distant knowledge, backward citation, technical field

## Abstract

This article is a modest attempt to shed some light on the question of linkages between backward and forward citations in technical fields posed by Trajtenberg et al. (1997). They found interesting similarities and high correlations between equivalent measures looking forward and backward. They also implied the linkage between distant backward and distant forward citations. There are several questions to be posed in applying their insights to Japanese patent applications, however, due to the differences in the patent classification system and the subject of citation, i.e., citations by the applicant or examiner, between the US and Japan. In addition, and most importantly, the possibility that subsequent classifications may match, even if the first classification is different, is unavoidable with existing measurement methods of technical distance. In order to investigate these research questions, the author proposes a new measurement method for the technological proximity between examiner’s citations and their originating patents using IPC-based patent classifications. Using such a proposed method, the author created two hypotheses and tested them for about 14,000 examined patent applications filed in 2008 with the JPO. As a result of testing Hypothesis I, the author confirmed that Trajtenberg et al.’s insights can be applied to Japanese patent applications using citations by the examiners and IPC-based patent classifications. In other words, it was confirmed that patent applications citing backward citations categorized in a technical field distant from the invention are more likely to be cited by forward citations categorized in a technical field distant from the invention. As a result of the verification of Hypothesis Ⅱ, it was further confirmed in some technical fields that the backward citations categorized in a technical field distant from the invention are more likely to be in the same technical field as the forward citations categorized in a technical field distant from the invention. The author believes that these verified results indicate the possibilities of using backward citations as a starting point from which we can find patent applications for inventions at an early stage with potential applicability to other technical fields.

## Introduction

This article is a modest attempt to shed light on the question of linkages between backward and forward citations posed by Trajtenberg et al. (1997), which is known as one of the leading studies of patent citations. In their paper, they aimed to demonstrate the potential usefulness of citations by comparing the citation rates of university patents and corporate patents. After constructing two sets of measures looking forward (“F measure”) and backward (“B measure”), they created such metrics as “diversity” and “technical distance” applied thereto. They called the diversity “generality” when applied to forward citation and “originality” when applied to backward citation (Jaffe, 2017, p1363). Although they expected that both measures would be larger for more basic inventions, their hypotheses were supported for generality, but not for originality. Later in their paper, on the other hand, they indicated another interesting result. They found similarities and correlations between equivalent measures looking forward and backward and suggested that research that draws from far removed technological areas leads to innovations of wider technological applicability (Trajtenberg et al., 1997, p46).

The author focuses on the latter finding, especially the linkage between distant backward and distant forward citations since it is considered to be very significant in that it implies that we can find patent applications for inventions with potential applicability to other technical fields at an early stage without relying on forward citations, which need a wide time window in order to get significant coverage [n1]. Jaffe (2017) points out that there are differences in citation practices due to institutional differences [n2] between the United States and other countries, including Japan (Ibid. p1360). The author describes two additional differences and one issue to be considered when applying their insights to Japanese patent applications.

First, there is a difference in the patent classification system between the US and Japan. Trajtenberg et al. (1997) use the US patent classification (“UPC”), which is unique to the US, as a measuring ruler whereas Japan uses the International Patent Classification (“IPC”), which is widely used in over 100 countries, to classify the content of patents in a uniform manner. Although US patent applications belong to about 400 main (three-digit) patent classes and over 120,000 patent subclasses, the UPC does not treat each three-digit patent class as roughly comparable in the ‘size’ of a technology (for example, the chemistry of inorganic compounds is a single class, whereas there are multiple optics classes) (Hall and Trajtenberg, 2004, p.4). The UPC is different from the IPC with regard to the hierarchical structure and size uniformity of the technology.

Second, there is a difference in the type of citation, i.e., the citation by the applicant or the citation by the examiner, between the US and Japan. The former refers to patent documents cited by the applicant (inventor) in the patent specification or the information disclosure statement, whereas the latter refers to patent documents cited by the examiner in the process of examination. In the United States, there are circumstances in which it is not possible to distinguish between the two on the database. This was the case until 2001, although it has been reported that the former type of citation tends to be used (Yasukawa, 2017, p. 74). On the other hand, in Japan, the citation information recorded in the database is the latter type of citation. While there are already many previous studies that positively support the usefulness of the former method of citation by the applicant, the evaluation of the usefulness of the latter method of citation by the examiner has been divided. Recently, however, there have been studies evaluating the usefulness of citation by the examiner as an indicator of patent evaluation (Yamada, 2010; Yasukawa and Kano 2014, Yasukawa, 2017).

Third, and most importantly, is the question of the metric of the technology distance. The measurement method proposed by Trajtenberg et al. (1997) is well known and used by many scholars, but, since the comparison focuses on the first classification, the problem remains that even if the first classifications are different from each other, it is not possible to distinguish the possibility of matching one of the subsequent classifications. This is a problem that could commonly occur in Japan as well as the US. As technology has become more complicated, there are many patent applications in which the two inventions differ in the first classification but match in the subsequent classifications, and further improvement has been sought [n3].

Jaffe (2017) pointed out that much of the empirical research relied on US citations, but he also mentioned there were important differences across jurisdictions in citation rules and practice, which creates interesting opportunities for research on non-U.S. data (Ibid. p.1360). Thus, the author tries to apply Trajtenbergs et al.’s insights to Japanese patent applications and explores the possibility of finding a patent application with potential applicability to other technical fields at an early stage by using backward citations as a starting point.

This article proceeds as follows: after reviewing the previous literature on measuring the technological distance, the author poses issues to be addressed in this paper and creates hypotheses in *Issues Addressed in This Article and Hypotheses Setting*. *Methodologies and Conceptual Model* summarizes the methodologies that make it possible to solve the issues and describes a conceptual model. *Data and Methods* describes the data used in this paper and describes a method for extracting data. In *Results*, after indicating the results of the analysis based on the data, the hypotheses are evaluated with a logistic regression analysis and table cross-sections. In *Discussion*, the author discusses the results of the evaluation, mentions some limitations, and suggests future research directions. Finally, the author presents the conclusions of this article in *Conclusion*.

## Issues Addressed in This Article and Hypotheses Setting

### Scope of This Article

Jaffe (2017) classifies research using patent citations into two broad groups (Ibid. p1361). One research line uses citations as an indicator of invention attributes in order to characterize the inventions. The other research line uses them as proxies for knowledge linkages across inventors in order to explore the nature of knowledge flows. This study is located in the former line and follows the approach of Trajtenberg et al. (1997) that characterizes both backward and forward citations. He also considers technical diversity as well as technological distance in order to characterize both citations which span the technological space defined by the classification scheme (Jaffe, 2017, p1363). In this paper, however, the author only focuses on the technology distance so as to concentrate on the linkage between backward and forward citations far from the original patent application.

### Measurement Method Proposed by Trajtenberg et al. (1997)

Trajtenberg et al. (1997) proposed a measurement method that uses a hierarchical structure of the U.S. patent classification system consisting of the three-digit patent classes, two-digit categories, and six very broad fields. This method is well known and used by many scholars. On the other hand, Hall and Trajtenberg (2004) pointed out that all of the generality measures suffer from the fact that they treat technologies that are closely related but not in the same class in the same way that they treat very distant technologies. This inevitably means that generality may be overestimated in some cases and underestimated in others (Ibid. p.20). This issue can be further divided into two.

The first issue is the lack of uniformity in the size of technology due to using the U.S. patent classification as a measure. Hall and Trajtenberg (2004) pointed out that the US patent classification does not treat each three-digit patent class as roughly comparable in the ‘size’ of a technology (for example, the chemistry of inorganic compounds is a single class, whereas there are multiple optics classes) (Ibid., p.4).

The second issue arises from the possibility of subsequent classes being in common. Since the comparison focuses on the first classification, the problem remains that even if the first classifications are different from each other, it is not possible to distinguish the possibility of matching one of the subsequent classifications [n4].

### Previous Literature on Measuring the Technological Distance

Several methods have been proposed for measuring technical distances based on patent classification [n5] other than Trajtenberg et al. (1997). Jaffe (1986) has been one of the most popular ways of measuring technological distances between firms. He used the classification symbols assigned to the firm to investigate the technical similarity of development between patent portfolios of firms. Akcigit et al. (2016) measure the technological propinquity between a patent and a firm by finding the number of all patents that cite patents from two different technology classes (two digits of its IPC code) simultaneously. Many researchers following Jaffe (1986) have defined different technical distance concepts using patent data for individual analytical purposes, many of which have been proposed to measure the proximity between firms (e.g., Rosenkopf and Paul, 2003; Benner and Waldfogel, 2008). All of these are excellent measurement methods, but it remains a problem that, because they use the primary patent class assigned to patents, patents classified in different primary patent classes may be in the same subsequent classes.

Jaffe (2017) points out that few studies focus on differences in technical fields related to the relevance of patent citation data and argues that citation indicators need to be verified and new indicators are developed (Ibid. p.1,371). Thus, the author addresses the first issue by using a version of the IPC controlled by another classification instead of using the IPC as is. The author also deals with the second issue by proposing a new methodology using the patent classifications of the patent applications and their backward citations. These will be described in more detail in *Methodologies and Conceptual Model*.

### Introducing Hypotheses

Here, the author would like to return to Trajtenberg et al. (1997) again. Based on the analysis results for “GENERAL” (corresponding to “F-measure”), they mention the following:

*“We turn now to a preliminary examination of the linkages between backward and forward measures that may throw light on issues related to the R&D process* […] *Thus it would seem that “importance breeds importance”, originality breeds generality, coming from far away in technology space leads far away as well, etc. In that sense, then, the (ex post) characteristics of patented innovations appear to be related to the attributes of the research that lead to them”* (Trajtenberg, et al., 1997, p.45).

The sentence mentioning “coming from far away in technology space leads far away” implies a linkage between distant backward and distant forward citations. As mentioned in *Scope of This Article*, Jaffe (2017) considers technical diversity as well as technological distance in order to characterize both citations which span the technological space defined by the classification scheme (Ibid. p1363). The author, however, considered that, as far as the linkage between distant backward and distant forward citations goes, it can be proved by relying on the technological distance only without investigating technical diversity. If their implications are applied to Japanese patent applications, using citations by the examiners and IPC-based patent classifications, it would be possible to predict the potential applicability of an invention to other technical fields at an early stage by using backward citations as a starting point. Thus, Hypothesis Ⅰ is created.

Hypothesis Ⅰ: The insights of the linkage between distant backward and distant forward citations posed by Trajtenberg et al. (1997) can be applied to Japanese patent applications using citations by the examiners and IPC-based patent classifications. In other words, the patent application for an invention that cites backward citations in a technical field distant from the invention tends to be cited by forward citations in a technical field distant from the invention.

On the other hand, Trajtenberg et al. (1997) suggest that there may be strong “family effects” in successive generations of patents (Ibid. p 47). Although the meaning of “family effects” is not necessarily definite, if the patent classification for distant technical field of backward citations and that for distant technical field of forward citations tend to match, we can not only find patent applications for inventions with potential applicability to other technical fields at an early stage but also find we may be able to discover a patent classification (i.e., technical field) to which the patent application for the inventions apply. Subsequently, Hypothesis II is created.

Hypothesis II: The backward citations categorized in a technical field distant from the invention are more likely to be in the same technical field as the forward citations categorized in a technical field distant from the invention.

If the results of the verification of these hypotheses are positive, it could be said that inventors and managers would be able to identify inventions (technologies) with high potential applicability in other technical fields at an early stage by investigating the technical fields of the prior art of the subject application as if analyzing DNA.

To evaluate the linkages between backward and forward measures in technical fields, the author proposes a new method that makes it possible to divide a set of patent applications into three categories consisting of “same”, “neighboring”, and “distant” technical fields depending on the proximity of the components of the invention to be combined. This new method can be used to examine whether an invention introducing technical elements from backward citations in the distant technical fields is more cited by forward citations in the distant technical fields. Although such an exploration might be made using data from a variety of countries, this article uses data of Japanese patent applications examined by the Japanese Patent Office (“JPO”) as examples.

### Indicators

As an indicator for evaluating Hypothesis Ⅰ, the ratio of the number of subject applications to the number of cited applications is calculated for each kind of backward citation and expressed as a percentage in accordance with the following equations:

Ratio of Subject Applications in Same-Field = (Number of Subject Application in Same Field / Number of Cited Applications) × 100 (%)

Ratio of Subject Applications in Neighboring-Fields = (Number of Subject Application in Neighboring Fields / Number of Cited Applications) × 100 (%)

Ratio of Subject Applications in Distant-Fields = (Number of Subject Application in Distant Fields / Number of Cited Applications) × 100 (%)

On the other hand, Hypothesis II examines the degree of coincidence between the patent classification given to the backward citation classified in the distant field and the patent classifications given to the forward citations, classified in the three technical fields. The comparison is made between the backward citation classified in the distant field and each of the three technical fields of the forward citations. The degree of coincidence is judged at the subclass level of the IPC. The first patent classification for the backward citation is compared with all patent classifications, including subsequent patent classifications for the forward citations. In this paper, the percentage of “degree of agreement” is referred to as “Matching Rate” and is defined for each technical field as follows.

Matching Rate in Distant Field means the result of the comparison between the backward citation classified in Distant Field and the forward citation classified in Same Field and is defined as follows:

Matching Rate in Distant Field = (Among the number of Citations defined in the denominator, the number of Citations whose technical fields match the backward citations categorized in Distant Fields / Number of Forward Citations in Distant Field) × 100 (%)

Matching Rate in Same Field and Matching Rate in Neighboring Field are defined analogously.

### Definition of Technical Terms Pertinent to the Model

Regarding patent applications and citations, “Subject application” (or “subject application”) is defined as a patent application filed in 2008 and examined by the JPO and citing at least one patent application as prior art. “Backward citation” is defined as a prior patent application cited by an examiner in the examination of the subject application. “Forward citation” is defined as a subsequent patent application citing the subject application in the examination. “Cited application” is defined as a patent application cited by one of the forward citations of a subject application whereas “non-cited application” is defined as a patent application not cited by any one of the forward citations of a subject application.

Regarding the three technical fields, “same field” (or “same-field”) is defined as a technical field that is the same as that of the subject application in the ITC’s Classes. “Neighboring Field” (or “neighboring field”) is defined as a technical field that differs from the ITC’s Class of the subject application but is the same as at least one of all IPC subclasses shown in the laid-open publication [n6] of the subject application. “Distant Field” (or “distant field”) is defined as a technical field that differs not only from the ITC’s Class of the Subject application but also from any one of all IPC subclasses shown in the laid-open publication of the subject application.

Regarding backward citations categorized into three technical fields, “Backward citation in same-field” is defined as a preceding patent application categorized in the same field. “Backward citation in neighboring-fields” is defined as a preceding patent application categorized in a neighboring field. “Backward citation in distant-fields” is defined as a preceding patent application categorized in a distant field.

Regarding forward citations categorized into the three technical fields, “Forward citation in same field” is defined as a subsequent patent application citing subject applications that all satisfy the condition of the same field. “Forward citation in neighboring field” is defined as a subsequent patent application citing subject applications of which at least one satisfies the condition of neighboring fields. “Forward citation in distant field” is defined as a subsequent patent application citing subject applications, all of which satisfy the condition of distant fields.

## Methodologies and Conceptual Model

This section summarizes the data analysis method based on the methodologies employed for evaluating the inventive step under the patent system [n7].

### Methodologies and Analysis Method

In this study, the author applies the concept of an “inventive step (non-obviousness)” used in the examination of inventions for a patent application [n8]. More specifically, the author pays attention to the possibility that one of the prior art documents cited in the examination is likely to differ in the technical field from an invention claimed in the patent application in the case that the invention incorporates technologies in other fields because it is assumed that one of the components of the invention is different in the technical field from the claimed invention [n9].

### International Patent Classification Controlled by Integrated Technology Classification as a Newly Proposed “Scale”

It is not easy, however, to decide what defines a difference in technical fields due to the difficulty of patent law, which requires examining a claimed invention from the viewpoint of the Skilled Person (Patent Act 29(2)). Thus, an existing patent classification system is used in this analysis as an alternative way of defining the technical field of an invention. The most widely used classification is the IPC, which is used in over 100 countries to classify the content of patents in a uniform manner [n10]. In this paper, on the other hand, another measuring ruler is used in combination with IPC. That is the IPC-based Integrated Technology Classification (“ITC”) (Goto and Motohashi, 2005), which has 33 technology classes (“ITC’s Classes”). There are two reasons for combining the ITC with the IPC. First, the ITC is aggregated into 33 technology classes, so it has an appropriate measurement range in that it is narrower than the section level of the IPC with eight sections and wider than the class level of the IPC. Second, by using the IPC as the classification controlled by the ITC, the “hierarchical structure” of the IPC can be brought closer to the current technology category.

The second reason is related to the relationship between the IPC and the ITC. For an English translation of the ITC-IPC comparison table, see Table 1 shown on page 1,433 of Goto and Motohashi (2007) with the title of “Aggregated technology classification. In this table, for example, “Pharmaceutical (IPC: A61K)” is included in Class A61 (Medical) in the IPC, but the ITC has made it independent as Class 5 (Drugs). “Genetic Engineering (C12N 15/00)” is included in Class 12 (Biochemistry, Enzymology) in the IPC, but is classified in Class 17 (Genetic Engineering) in the ITC [n11]. In each case, there is an effort to get closer to the real technology category.

**TABLE 1:** Cross table showing the relationship.

			F-same	F-neighbor	F-distant	Total	
Backward Citation	B-Same	①	3,628	1,359	744	5,731	Backward Citation
		②	63.30	23.71	12.98	100.00	%
		③	84.83	70.31	71.61	79.06	%
	B-Neighbor	①	456	465	185	1,106	
		②	41.23	42.04	16.73	100.00	%
		③	10.66	24.06	17.81	15.26	%
	B-Distant	①	193	109	110	412	
		②	46.84	26.46	26.70	100.00	%
		③	4.51	5.64	10.59	5.68	%
	Total	①	4,277	1,933	1,039	7,249	
		②	59.00	26.67	14.33	100.00	%
		③	100.00	100.00	100.00	100.00	%

Pearson chi2 (4) = 260.0126 Pr = 0.000 Likelihood-ratio chi2 (4) = 244.3888 Pr = 0.000 Cramer's V = 0.1339 gamma = 0.3132 ASE = 0.021 Kendall's tau-b = o.1522 ASE = 0.011.

### Method for Measuring the Technological Proximity Between a Patent Application and its Citations

In this sub-section, a method for measuring the technological proximity between the patent application and its citations is described. This is made possible by using, as well as the IPC controlled by the ITC, the IPC system having subsequent classes allocated to a patent application together with the first class. From the explanation mentioned below, it will be understood that the different types of inventions can be explained by the comparative relationship between patent applications for an invention examined by an examiner (the “subject application”) and backward citations cited by an examiner in the examination of the subject application.

**FIGURE 1:**
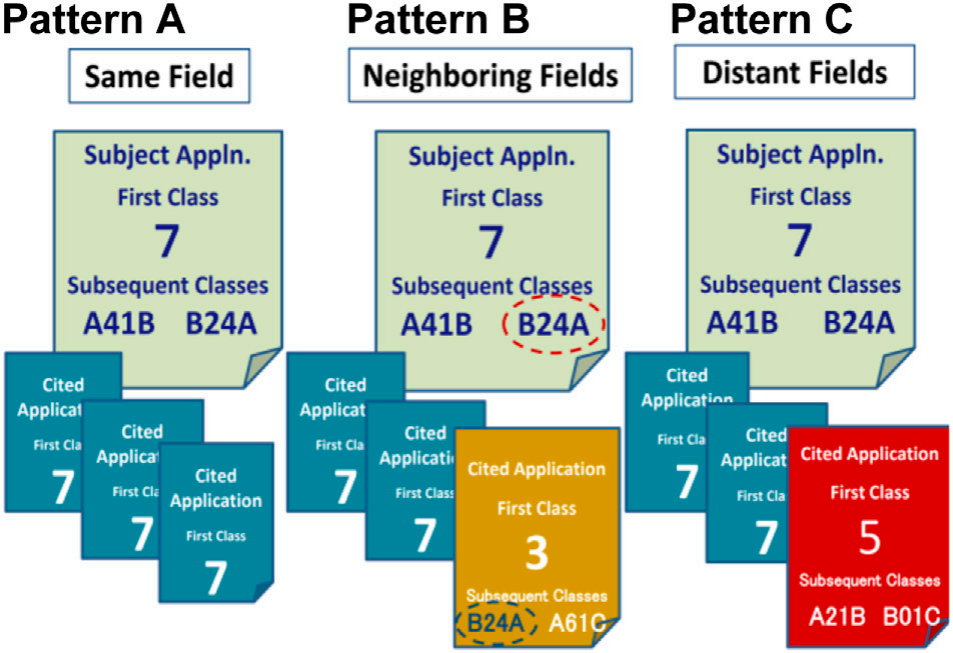
Three patterns of examined patent applications based on technical fields in backward citations.

Looking at Figure 1, one can see there are three patterns, A to C, of subject applications categorized in Class 7. They are cited by several backward citations categorized in Class 3, 5, and 7. Pattern A shown in Figure 1 is different from the other patterns in that all of the backward citations are in the same category as the subject application in the ITC (“coincidence”). The subject application is categorized in Class 7, and it agrees with all backward citations also being categorized in Class 7. On the other hand, Pattern B shown in Figure 1 and Pattern C shown in Figure 1 are similar in that at least one of the backward citations is different from the subject application in the ITC (“non-coincidence”). Patterns B and C categorized in Class 7 do not agree with one of the backward citations, which are categorized in Class 3, shown in Pattern B of Figure 1, as well as Class 5, shown in Pattern C of Figure 1.

Based on a comparison of subject applications and backward citations using the ITC, Pattern A can be defined as a patent application for an invention including backward citations, all of which satisfy the condition of coincidence in the ITC (same-field). On the other hand, Patterns B and C can be defined as a patent application for an invention including backward citations, at least one of which satisfies the condition of non-coincidence in the ITC. And thus, Patterns B and C of the subject applications can be distinguished from same-field applications with the use of backward citations.

### First Class and Subsequent Classes

The next step is how Pattern C can be distinguished from Pattern B. In order to achieve this, the author focuses on the IPC system which has the subsequent classes allocated to a patent application together with the first class.

As shown in Pattern A of Figure 1, it can be seen that even if a subject application is different from backward citations at the level of the ITC’s Class corresponding to the first class of the IPC, there is a possibility that the subject application has close proximity with backward citations at the level of subsequent classes of the IPC. More concretely, as shown in Pattern B of Figure 1, a subject application categorized in ITC’s Class 7 is different from its backward citation as far as looking at ITC’s class 3, but it is categorized in the same field as the backward citation when comparing the subsequent classes of the IPC classified in B24A. This is because the ITC corresponds to only the first class among several IPC classes allocated to a patent application.

On the other hand, as shown in Pattern C of Figure 1, a subject application categorized in ITC’s Class 7 is not only different from its backward citation categorized in ITC’s Class 5 but also different from its backward citations even when comparing with the subsequent classes of the IPC. The subsequent classes of the subject application with A41B and B24A do not match any of the subsequent classes of its backward citation with A21B and B01C. That is that the two patterns B and C shown in Figure 1 are common in that both patterns have at least one backward citation different in the ITC from that of their Subject Application. However, they are different from each other in that the subject application of Pattern B shown in Figure 1 is coincident with at least one of the backward citations at the level of the subsequent class (“neighboring-fields”), whereas the subject application of Pattern C shown in Figure 1 is different from any backward citations at the level of the subsequent class as well as the level of the first class (“distant-fields”). Thereby, the two patterns can be distinguished from each other.

From the explanation above, it is understood that the data of patent applications belonging to neighboring fields are extracted by deducting the data of patent applications belonging to distant fields from the data of patent applications categorized in non-coincidence. It is also understood that an invention whose subject application is classified in distant fields is further apart from an invention classified in the same field. Based on the above-mentioned methodologies using backward citations cited by an examiner, it is understood that patent applications filed in a given period can be classified into three groups consisting of same, neighboring, and distant technical fields.

Although the above-mentioned analysis method is explained by the relationship between a subject application and the backward citations thereof, the same method can be applied to the relationship between a subject application and subsequent patent applications citing the subject application in the examination (“forward citations”).

### Conceptual Model

**FIGURE 2:**
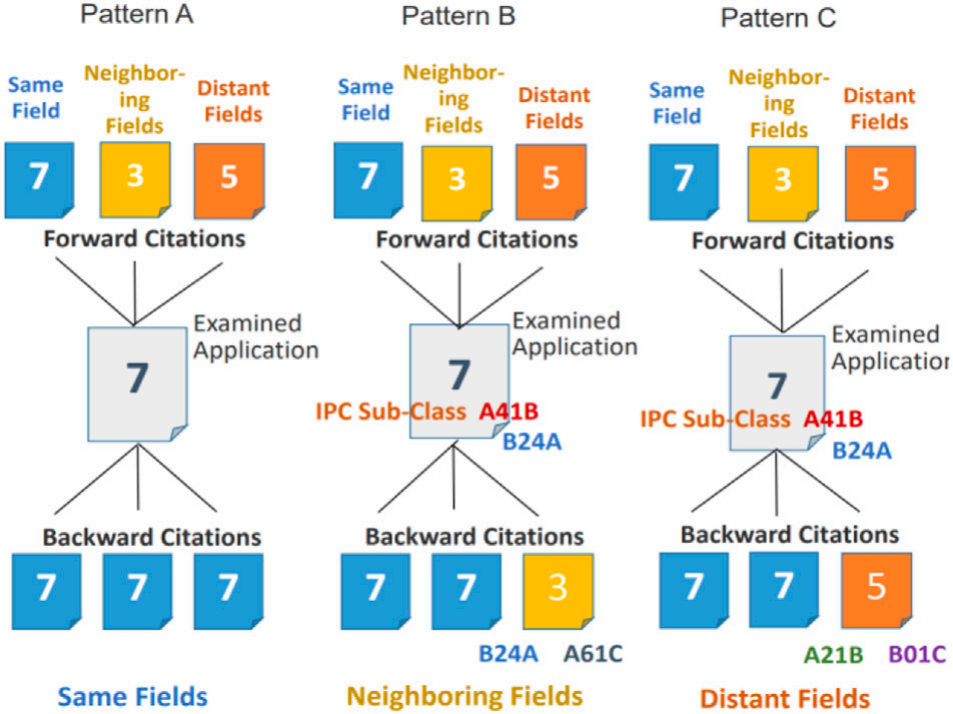
Conceptual model showing the relationship between backward citations and forward citations.

In the above description, three kinds of subject applications are explained based on Figure 1, indicating three types of subject applications categorized in accordance with technical fields in backward citations. Figure 2 is a conceptual model which adds the relationship with forward citations to Figure 1 and shows the relationship between backward citations cited by three types of subject application and forward citations citing three types of the subject application. The former is illustrated in the lower half of Figure 2, whereas the latter is illustrated in the upper half thereof. In the center of the figure, three types of subject applications are illustrated as same, neighboring, and distant fields from the left to the right. Along with each pattern of the subject application, three types of backward citations are exemplified in the lower half as applications belonging to same-, neighboring-, and distant- Fields, whereas three types of forward citations are exemplified in the upper half as well.

From the explanation mentioned in previous paragraph, it is understood that forward citations can be categorized into three kinds of technical fields as well in accordance with the technical fields of Subject applications. Three types of forward citations are shown in the upper half of Figure 2 as Patterns A, B, and C. Taking pattern A as an example, the three kinds of forward citations are exemplified and labeled as “forward citation in same field,” “forward citation in neighboring fields” and “forward citation in distant fields” from the left to the right.

According to the proposed method, it becomes possible to analyze the relationship of technical fields between backward citations and forward citations. More concretely, by checking which of the three kinds of subject applications tends to be cited by which field of forward citations, an answer for the research question posed in the introduction can be obtained.

## Data and Methods

### Data

In this study, the research was conducted on Japanese patent applications filed in 2008. The filing year of 2008 refers to the actual filing year of the subject application. And thus, the filing year based on the priority date or the filing date of the patent application is not included. In the case of an international application, the year of the international filing date is adopted. The reason for choosing the applications filed in 2008 is that the year 2008 is considered to be the latest year for which the Japanese Patent Office has completed most of the examinations requested under the examination-on-demand system as of August 31, 2017, on which date the data were extracted from databases.

Among the patent applications filed in Japan in 2008, the total number of applications for which examination was requested and the results were obtained on the above date of data extraction is 235,078. Of these, the six technology classes 1, 13, 14, 20, 26, and 30, with similar numbers of IPC subclasses from the 33 ITC technology classes were selected for this search. The reason why these six classes were selected is to match the conditions of the technical fields by comparing the target data in which the numbers of IPC subclasses are close to each other. The total number of the six technology classes is 14,114, and the number of subject applications for each class is shown by class in Column X (i) in Supplementary Appendix Table S1.

Part of the data was obtained by comparing the ITC Class of subject applications with the ITC’s Class of backward citations using the citation information of the IIP Patent database with MySQL on August 31, 2017 [n12], whereas the other data were extracted from some databases including data of patent classifications, applicants and examination results. These data were merged into one set of data tables using the application number as a common key. Prior art documents, which are stated as a reference in the official action but are not cited as the basis for the rejection, are also included in both forward and backward citations in this study [n13]. Due to database restrictions, this study was limited to patent applications and does not include utility model patent applications.

### Method for Extracting Data

This sub-section describes a method for extracting data, proposed in this article, by comparing subject applications with backward citations through two filtering processes with technical classifications in different levels, the details of which are described below.

#### First Filtering Process

**FIGURE 3:**
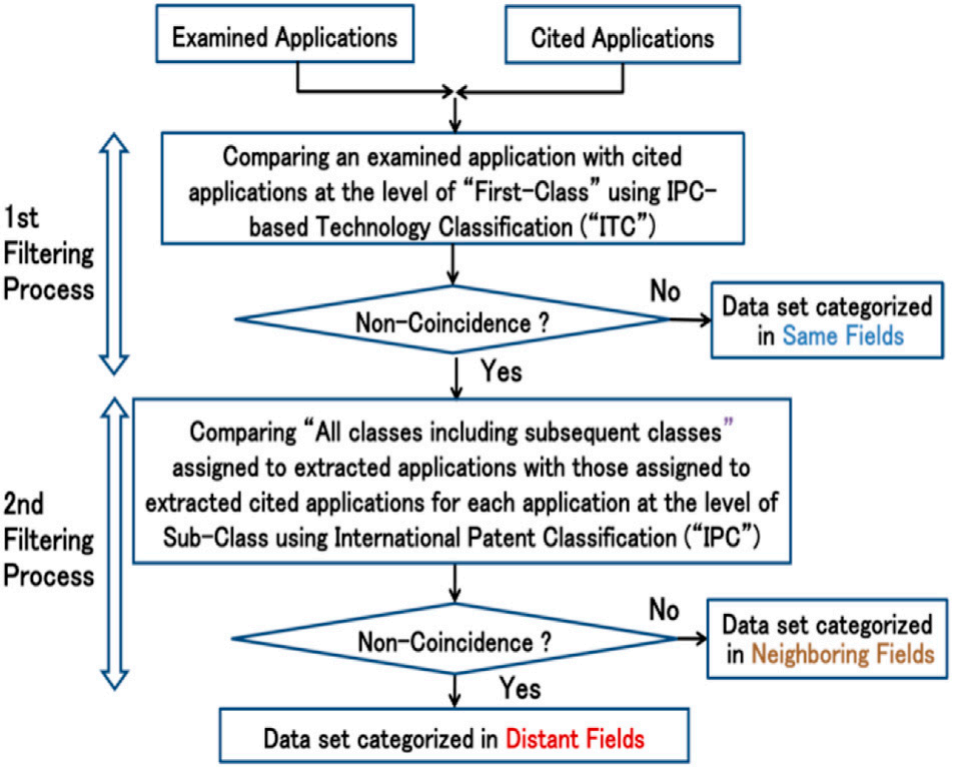
Flow chart of two-step filtering processes.

Initially, the first filtering process is performed, as shown in the upper half of Figure 3, by identifying subject applications which are different from backward citations thereof in the ITC’s Class of each subject application. These identifications are, as shown in Figure 3, conducted by comparing subject applications with backward citations at the level of “first class of IPC” using the ITC. In the research for this article, the first filtering process was conducted for examined patent applications filed in 2008, for the reasons mentioned in *Data* above.

Following the comparison at the level of the first class, data are separated into two groups. That is, if there is at least one backward citation whose ITC’s Class is different from the ITC’s Class of its subject application, the data is determined as non-coincidence, On the other hand, if there are no backward citations whose ITC’s Class is different from the ITC’s Class of their subject application, the data is determined as coincidental, which means that the subject application is categorized in the same field. In this way, subject applications consisting of elements in the same field are separated from subject applications potentially including elements in distant fields as well as neighboring fields.

#### Second Filtering Process

Next, the second filtering process is conducted for the set of applications extracted in the first filtering process. The second filtering process was performed, as shown in the lower half of Figure 3, by comparing all IPC sub-classes, including subsequent classes, assigned to the extracted subject applications with those assigned to backward citations for each subject application. This comparison was performed by downloading the IPC subclass data into Excel from a general patent database.

After the comparison of IPC sub-classes, data extracted from the first filtering process are separated into two groups. That is, if at least one of all subsequent classes assigned to the subject applications is in agreement with any one of all subsequent classes assigned to the backward citations, the application is determined as coincidence, which means that the application is categorized in “neighboring fields”. On the other hand, if there is no coincidence among all subsequent classes between subject applications and backward citations, the application is determined as non-coincidence, which means that the application is categorized in “distant fields”. In this way, a set of the applications categorized in distant fields is separated from the data categorized in neighboring fields.

#### Categorization of Backward Citations

Through the two filtering processes described above, examined patent applications filed in 2008 are categorized in three kinds of technical fields consisting of same, neighboring, and distant fields. The number of subject applications for the technical fields above is 180,382, 36,944, and 17,752, respectively, as can be seen in the row titled “6 Totals” in Supplementary Appendix Table S1.

#### Categorization of Forward Citations

As explained in *Conceptual Model*, the analysis method described above can be applied to the relationship between subject applications and forward citations as well. More concretely, subsequent applications citing subject applications can be categorized through two filtering processes into the three kinds of technical fields in accordance with the definitions mentioned in *Definition of Technical Terms Pertinent to the Model*. Then, after they are collated, it is confirmed which field of the subject application is cited by which field of the forward citations.

As the result of the collation, subject applications are categorized into two groups, which are applications cited by forward citations (“cited applications”) and applications not cited by forward citations (“non-cited applications”). The total number for each of the two groups is shown in column X (ii) and column X (iii) in Supplementary Appendix Table S1, respectively. Furthermore, cited applications are categorized into three technical fields for each selected field of the ITC. The number and the ratio of subject applications cited by forward citations in each of three technical fields are shown in Column Y and Column Z in Supplementary Appendix Table S1, respectively. For example, the ratio of subject applications cited by forward citations in distant fields is shown for each technical field of backward citations in column Z (x) as a greyed-out area.

## Results

This section explains the results of the data analysis for each hypothesis.

### For Hypothesis Ⅰ

**FIGURE 4:**
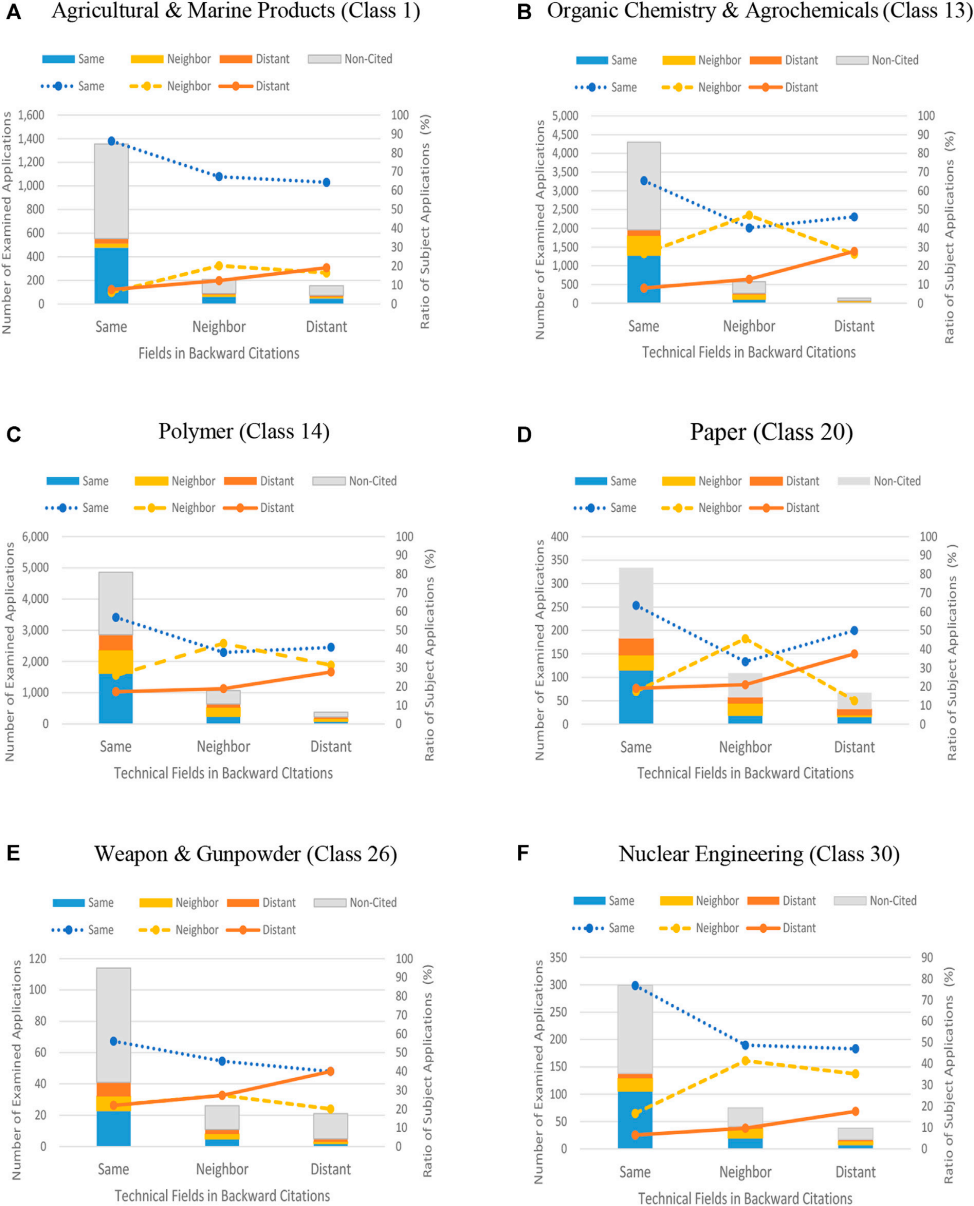
Graphs showing the relationship shown in Table 1 for each selected class of ITC. Notes: Figures 4A-F are graphs showing data for each ITC class below. A: ITC Class 1 (Agricultural and Marine Products), B: ITC Class 13 (Organic Chemistry and Agrochemicals), C: ITC Class 14 (Polymer), D: ITC Class 20 (Paper), E: ITC Class 26 (Weapon and Gunpowder), F: ITC Class 30 (Nuclear Engineering). On the graphs for each technical field, the ratio of subject applications as well as the number thereof are displayed. The number of examined subject applications, corresponding to the left vertical axis, is indicated by bars classified by color on the basis of the difference of values for three technical fields in forward citations. Whereas, the ratio of subject applications, corresponding to the right vertical axis, is indicated by lines classified by color, and the kind of lines according to a prescribed calculation formula of the ratio of subject applications on the basis of the difference of values for the three technical fields in forward citations.

Data showing the relationship between the backward citations and the forward citations for the six selected technology classes of the ITC are shown in Supplementary Appendix Table S1. Figures 4A–F are graphs representing the data shown in Supplementary Appendix Table S1 according to each ITC’s Class. As is evident from the graphs, the number of subject applications cited by forward citations gradually decreases toward the level of distant fields from the same field in backward citations. Similar patterns are observed among each of the technical fields in forward citations. Subsequently, a comparison is made between technical fields in forward citations by rearranging the acquired data according to the three technical fields in order to find if there is a tendency for similar patterns to occur.

**FIGURE 5:**
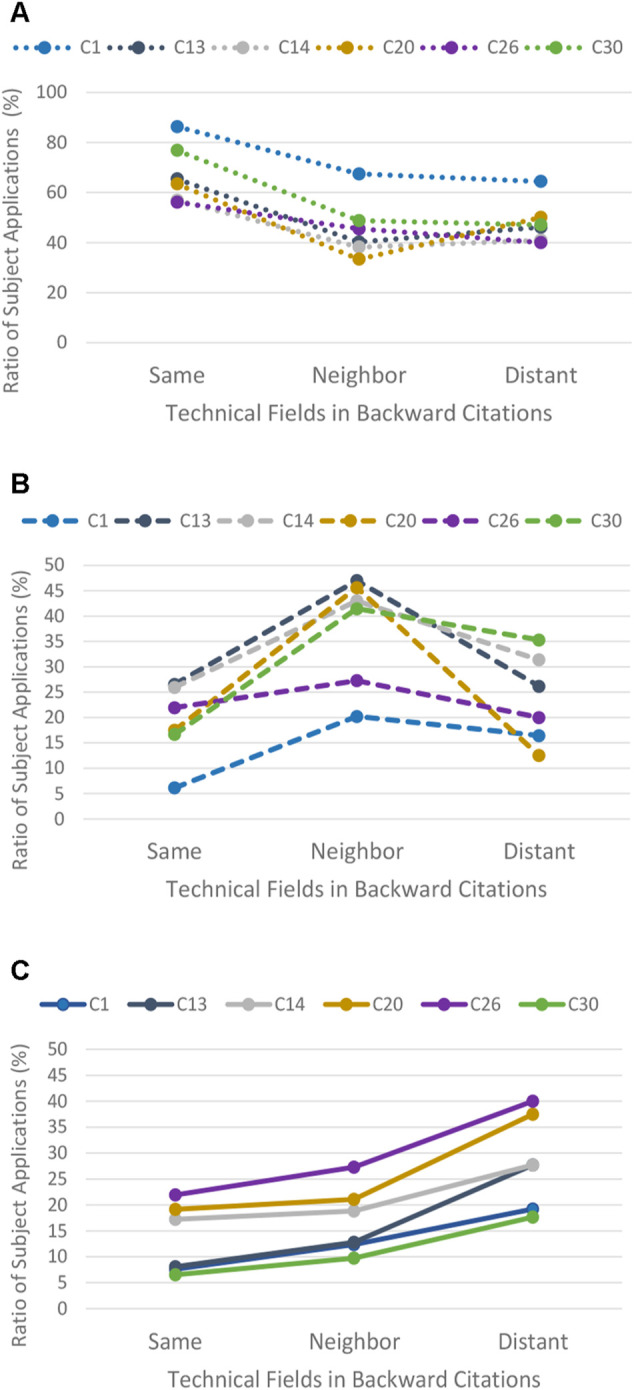
**(A)** Ratio of subject applications in “Same-Field” cited by forward citations in “Same Fields”. **(B)** Ratio of subject applications in “Neighboring-Field” cited by forward citation in “Neighboring Field”. **(C)** Ratio of subject applications in “Distant-Field” cited by forward citations in “Distant Fields”.

Figures 5A–C are line graphs indicating the ratio of subject applications for each of the three technical fields of forward patent citations. Each ratio is indicated along the vertical axis and is classified by the same kind of lines as those shown in Figures 4A–F. It is clearly shown in Figures 5A–C that there is a tendency of similar patterns coexisting in the inclination of line graphs in each technical field. In Figure 5A, showing same field in the forward citations, there is a tendency of a gradual decrease in the ratio with shifting from the same field to the distant field. On the other hand, in Figure 5C, showing distant fields in the forward citations, there is a tendency of gradual increase in the ratio of subject applications with shifting from the same field to distant fields in backward citations. In Figure 5B, showing neighboring fields of forward citations, there is a tendency of gradual increase in the ratio of Subject applications with shifting from the same field to neighboring field in backward citations, whereas there is the opposite tendency of a gradual decrease in the ratio of subject applications with shifting from neighboring to distant fields.

As is clear from comparing the three patterns shown in Figures 5A–C, the inclination of subject applications in distant fields shown in Figure 5C seems to be in good agreement with Hypothesis Ⅰ. That is, a glance at Figure 5C reveals that subject applications citing backward citations which are farther away from the subject application in technical fields are cited by forward citations, which are farther away from the subject application in technical fields. The data shown in Figure 5C is then evaluated with statistical analysis in the next section.

### For Hypothesis Ⅱ

Next, the results of the data analysis of Hypothesis II are explained. Supplementary Appendix Table S2 shows the degree of coincidence between the patent classification given to the backward citation classified in the distant field and the patent classifications given to the forward citation classified in the three technical fields of the forward citations. The results in Supplementary Appendix Table S2 show that each Matching Rate for the four ITC classes 01, 13, 14, and 20 is higher in neighboring fields than in the same field and distant fields than in neighboring fields, respectively. On the other hand, for the remaining two ITC classes 26 and 30, the number of matches was relatively small and the Matching Rate could not be calculated. In the following analysis, therefore, the four ITC classes other than 26 and 30 are examined.

**FIGURE 6:**
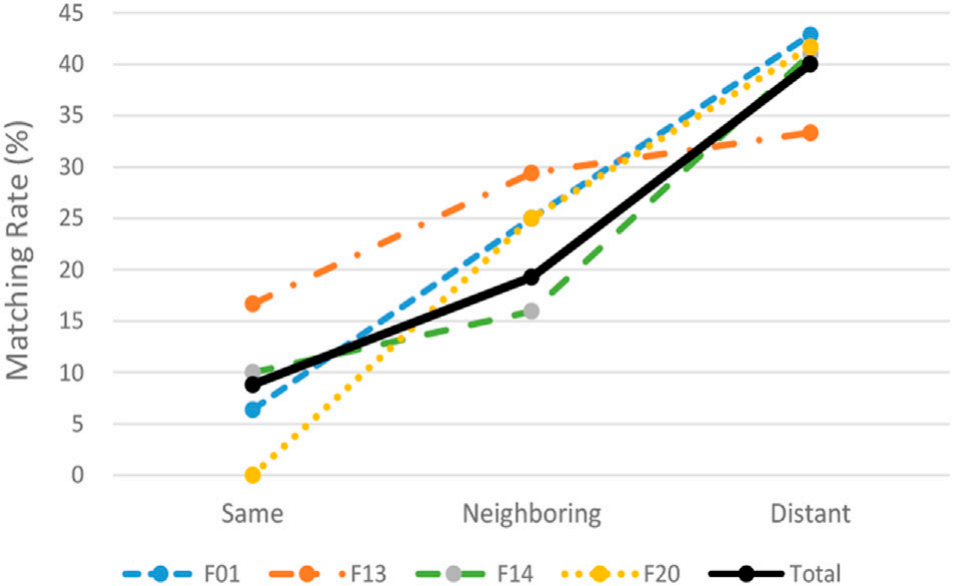
Line graphs showing relationship between the matching rates and the three technical fields.

Figure 6 contains line graphs showing the relationship between the Matching Rates and the three types of technical fields for the four ITC classes, 01, 13, 14, 20, and the total thereof. The lateral axis of the graph shows three technical fields consisting of same, neighboring and distant fields. The vertical axis represents the Matching Rate as a percentage. The Matching Rate based on the total value of the four technical fields increases as the technical field shifted from the same field to the distant field. The same tendency can be seen in all four ITC classes shown in Figure 6.

## Discussion

In this section, Hypotheses Ⅰ and Ⅱ are evaluated with logistic regression analysis and by looking at table cross-sections.

### Evaluating the Hypotheses

#### For Hypothesis Ⅰ

##### Analysis Based on Table Cross-Sections

First, the author will proceed with the verification of Hypothesis Ⅰ. Table 1 shows the relationship between the three technical fields of backward citations lined up vertically and those of forward citations lined up horizontally. In each column, upper, middle, and lower sub-columns indicate 1) the number of subject applications cited by forward citations in a given technical field, 2) the percentage share of the subject applications against total applications in a given technical field of a backward citation, and 3) another percentage share of the subject applications against total applications in a given technical field of a forward citation, respectively. It is understood from the cells highlighted in red that a column at an intersection point between the vertical line and the horizontal line in which a technical field agrees with one another indicates the largest percentage among the ratios of subject applications shown in the middle columns.

**FIGURE 7:**
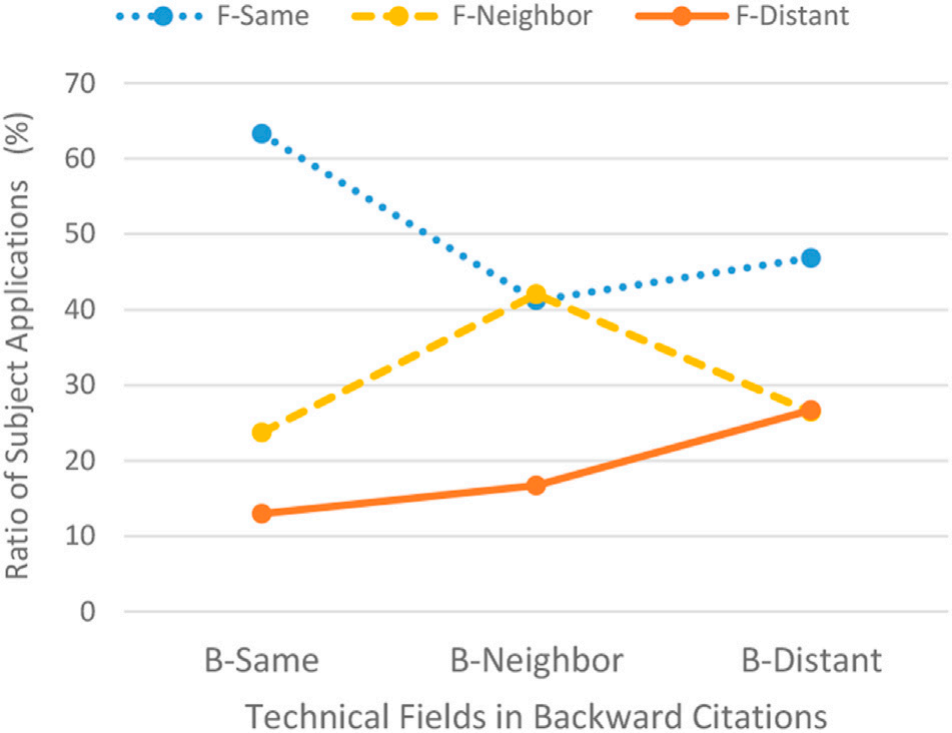
Line graph showing change of ratio.

The data for the ratio of subject applications shown in the middle sub-columns indicated in the above (2) are made into graphs as shown in Figure 7, which indicate the ratio of the subject applications on the vertical axis and the three technical fields of backward citations on the horizontal axis. The three technical fields of the forward citations are represented by three colored lines. Special attention should be paid to the orange-colored line which indicates a change in the ratio of patent applications categorized in distant fields among forward citations. As shown in Figure 7, the orange-colored line gradually increases from same-field (12.98%) to neighboring fields (16.73%) and to distant-fields (26.70%) for backward citations. The ratio for distant fields is as much as double that of same field.

##### Evaluation of Hypothesis Ⅰ With Logistic Regression Analyses

After obtaining these results, logistic regression analyses were conducted on the basis of the data in Figure 5C, focused on the change in the ratio of subject applications of patent applications categorized in distant fields among forward citations.

Table 2 summarizes the variables used in the analysis of Figure 5C. Table 3 shows the results of the logistic regression performed for data shown in Figure 5C. These logistic regressions are executed for the ratio of subject applications cited by forward citations in distant field as the explained variable and for two technical fields as the explaining variable. The two variables consisting of neighboring field (F-Neighbor) and distant field (F-Distant) are indicated on the comparison with same field.

**TABLE 2:** Summarizing variables.

Variables	(1)	(2)	(3)	(4)	(5)
	N	Mean	Sd	Min	Max
ITC_13	7,249	0.316	0.465	0	1
ITC_14	7,249	0.513	0.5	0	1
ITC_20	7,249	0.0375	0.19	0	1
ITC_26	7,249	0.00786	0.0883	0	1
ITC_30	7,249	0.027	0.162	0	1
Forward_C	7,249	1.553	0.731	1	3
F_Neighbor	7,249	0.267	0.442	0	1
F_Distant	7,249	0.143	0.35	0	1
B_Neighbor	7,249	0.153	0.36	0	1
B_Distant	7,249	0.0568	0.232	0	1

**TABLE 3:** Result of logistic regression.

Variables	F_Neighbor	F_Distant
B_Neighbor	0.867***	0.225**
	(0.0697)	(0.0907)
B_Distant	0.272**	0.845***
	(0.119)	(0.121)
ITC_13	1.470***	0.0589
	(0.14)	(0.149)
ITC_14	1.423***	0.801***
	(0.137)	(0.136)
ITC_20	1.027***	0.963***
	(0.197)	(0.197)
ITC_26	1.052***	1.160***
	(0.346)	(0.336)
ITC_30	1.075***	−0.155
	(0.216)	(0.292)
Constant	−2.493***	−2.413***
	(0.133)	(0.131)
Pseudo R2	0.0374	0.0303
Log Likelihood	−4,046.6877	−2,888.6687
Observations	7,249	7,249

Standard errors in parentheses ***p < 0.01, **p < 0.05, *p < 0.1.

As understood from Table 3, the coefficient of subject applications in neighboring fields (B-Neighbor) cited by forward citations in distant fields (F-Distant) is 0.225 times higher than that of subject applications in the same field cited by forward citations in distant fields at the level of 0.05% significance. Whereas, the coefficient of subject applications in distant fields (B-Distant) cited by forward citations in distant fields (F-Distant) is 0.845 times higher than that of subject applications in the same field cited by forward citations in distant fields at the level of 0.01% significance. Each coefficient is a positive value and the coefficient of distant fields is higher than that of neighboring fields. From these results, it can be confirmed that the ratio of subject applications cited by forward citations in distant fields increases with shifting backward citations from the same field to distant fields. The same tendency is indicated in other technical fields including ITC’s Classes 13, 14, 20, 26, and 30 at the level of 0.01% significance. Based on the results of the logistic regressions mentioned above, Hypothesis Ⅰ is supported.

#### For Hypothesis Ⅱ

Next, Hypothesis II is tested. Table 4 shows the results of logistic regression analyses for four ITC classes, 01, 13, 14, and 20, and the total thereof. Table 5 shows the summary of the variables used therein.

**TABLE 4:** Logistic regression estimation of four technical fields and total thereof.

	Total	F01	F13	F14	F20
VARIABLES	Result	Result	Result	Result	Result
Neighbor	0.868**	1.587*	0.734	0.535	16.97***
	(0.356)	(0.895)	(0.723)	(0.481)	(1.295)
Distant	1.873***	2.398***	0.916	1.833***	17.74
	(0.323)	(0.805)	(0.7)	(0.437)	(0)
Constant	−2.279***	−2.686***	−1.609***	−2.197***	−18.07***
	(0.255)	(0.597)	(0.49)	(0.351)	(0.586)
Observations	390	73	65	220	32

Standard errors in parentheses ***p < 0.01, **p < 0.05, *p < 0.1.

**TABLE 5:** Summary of variables.

Variable	Obs	Mean	Std. Dev	Min	Max
Result	390	0.2,025,641	0.4,024,266	0	1
Neighbor	390	0.2,615,385	0.440,037	0	1
Distant	390	0.2,692,308	0.4,441,299	0	1
Whole	390	1	0	1	1
F01	390	0.1,871,795	0.3,905,566	0	1
F13	390	0.1,666,667	0.3,731,567	0	1
F14	390	0.5,641,026	0.4,965,108	0	1
F20	390	0.0820,513	0.2,747,954	0	1

The results shown in Figure 6 are also reflected in the results of logistic regression analyses shown in Table 4. As shown in the “Total” column of the four ITC classes, the coefficients for both Neighboring and Distant Fields are positive relative to the same field at a level of significance of 5% for neighboring field and 1% for distant field, respectively. As for the ITC classes of 01 and 14, the coefficients for the distant field are positive relative to the same field at a level of significance of 1%. As for the ITC classes of 01 and 20, the coefficients for the neighboring field are positive relative to the same field at levels of significance of 10 and 5%, respectively. These results mean that backward citations categorized in a distant field are more likely to be in the same field as the forward citations categorized in a distant field than forward citations categorized in the same or neighboring fields. Therefore, these results support Hypothesis II.

### Examples of Actual Applications

A total of seven applications from the data analyzed for hypothesis verification are described below as examples. All cases are focused on patent applications citing the backward citation categorized as distant field and cited by forward citations categorized as the distant field.

Case 1: Patent application No. 2008–234092 (IPC: A01G), which relates to “base paper for fruit bags” having excellent strength and a water repellent finish, was cited by the forward citation No. 2011–165694 (IPC: D21H) relating to “mill wrapper paper for packaging pulp sheets” as a reference. One of the application's backward citations (e.g., No. 1985–143605) was consistent with its forward citation at IPC subclass D21H (pulp composition). The document indicating the backward citations of this application was published by the JPO about 4 years earlier than that of the forward citations.

Case 2: Patent application No. 2008–188625 (IPC:C08L) relates to a “higher-order structural change indicator” capable of detecting a change in a higher-order polymer structure based on a change in the hue. This application was cited by three forward citations categorized as distant field. Among them, patent application No. 2010–110907 (IPC: G09F) relates to a “label with peeling detection function,” which cannot be visually confirmed when the label is replaced but can be confirmed only by a specific person concerned that the label has peeled off. One of the backward citations of the subject application is given as G11B (information record) in the IPC. This is different from the G09F (display, symbol) given to the forward citation but is common in ITC class 29 (display, information record), which includes these IPC classes. The document showing the backward citations of this application was published by the JPO about 1.5 years earlier than that of the forward citations.

Case 3: Patent application No. 2008–183749 (IPC: C08G) relates to an “epoxy composition” having excellent thermal stability and low thermal coloring. This application was cited as the primary reference by the forward citation No. 2013–090354 (IPC: B01J), categorized as distant field, which relates to a method to easily and efficiently separating and recovering an oxoacid catalyst used in a reaction for oxidizing an organic compound by hydrogen peroxide [n14]. Although both applications are in different IPC subclasses, B01J (Chemical or Physical methods, e.g., catalysts and colloid chemistry) given to the forward citation is the same as the IPC subclass given to the two backward citations of the present application, and thus it is conceivable that the two technical fields are closely related technologies. The document showing the backward citations of this application was published by the JPO about 4 years earlier than that of the forward citations.

Case 4: Patent application No. 2008–120828 (IPC: C07D) relates to “a sulfonium salt, a photoacid generator and a curable composition” having photosensitivity to actinic radiations such as visible light, ultraviolet rays, and X rays. This application was cited as the primary reference by forward citation No. 2011–021767 (IPC: G03F) categorized as distant field, which relates to a “liquid discharge apparatus for generating an ink droplet used in an ink jet recording method”. Although both applications are different in IPC subclasses, G03F (manufacture of uneven or patterned surfaces by the photomechanical method), which is given in the forward citation, is the same as one of two backward citations of the present application, and thus it is conceivable that the two technical fields are closely related technologies. The document showing the backward citations of this application was published by the JPO about 2 years earlier than that of the forward citations.

The above four cases (1–4) all support Hypothesis II. They show that some of the backward citations categorized in a technical field distant from the invention are in the same technical field as the forward citations categorized in a technical field distant from the invention. Next, cases 5 to 7 are described.

Case 5: Patent application No. 2008–257926 (A01G) relates to “work support tools for viticulture, etc.”, which is able to balance at least one arm of the worker by elastic force and support the worker in a lifted state. This application was cited by four forward citations categorized as distant field. Among them, patent application No. 2015–525523 (B25J) was related to an “adaptive arm support system” used in surgery, dentistry, painting, dishwashing, and product assembly. The document showing the backward citations of this application was published by the JPO about 5 years earlier than that of the forward citations.

Case 6: Patent application No. 2010–508294 (WO 2008–140210) (IPC: B31B) relates to a “soft X-ray light ionizing charger”, which neutralizes particles contained in an aerosol by irradiation of soft X-rays. This application was cited as a primary reference by forward citation No. 2014–517083 (IPC: B09B) categorized into Distant Field, which relates to a “powder conveying system” capable of safely maintaining the facility by eliminating the danger of explosion due to static electricity. The document showing the backward citations of this application was published by the JPO about 4 years earlier than that of the forward citations.

Case 7: Patent application No. 2008–204273 (B32B) relates to “a gas barrier material containing cellulose fibers” having an average fiber diameter of not larger than 200 nm. This application was cited by seven forward citations categorized as distant field. They are divided into two groups depending on whether their IPC subclasses are shown in any of the backward citations. Among those shown in the backward citation were applications relating to a pulp composition (D21H), whereas the applications not shown includes applications relating to “a composite composition comprising a fibrous filler” (B82B), “a porous body comprising cellulosic nanofibers” (D04H), and “a water-based coating composition” (C09D). The documents showing the backward citations of the latter group of the applications were published by the JPO about 2 years earlier than that of the forward citations [n15].

The above seven cases all support Hypothesis Ⅰ. They show that patent applications citing backward citations categorized in a technical field distant from the invention are more likely to be cited by forward citations categorized in a technical field distant from the invention.

### The Academic Value and the Practical Implications of the Present Study

#### The Academic Value

As a result of testing Hypothesis I, the author confirmed that Trajtenbergs et al.’s insights, i.e., the linkage between distant backward and distant forward citations, are applicable to Japanese patent applications using examiner citations and IPC-based patent classifications. As a result of the verification of Hypothesis II, it was further confirmed in some technical fields that the backward citations categorized in a technical field distant from the invention are more likely to be in the same technical field as the forward citations categorized in a technical field distant from the invention.

#### The Practical Implications

The author believes that this study provides inventors and managers with insights into exploring technologies that may be applied in distant technical fields or in developing a new application of an invention.

From the above seven cases and the verification results of Hypothesis I, it is understood that, in order to find a patent application for an invention whose forward citations are likely to appear in a technical field far from the invention in the near future, we should first find a patent application that cites a backward citation in a technical field far from the present invention.

It is also understood from the above cases 1 to 4 and the verification results of Hypothesis Ⅱ, in order to find a technical field in which forward citations are likely to be filed in the near future, we should look at the technical fields of the backward citation after finding a patent application for an invention having backward citations in a distant field.

In addition, the recent increasing use of the Accelerated Examination System (“AES”) may further increase the potential of the new methodology in this article [n16]. Patent examination has been partially accelerated after the AES was introduced by the Japanese Patent Office [n17]. Systems similar to the AES have also been introduced in Germany and other major countries. This tendency will further increase the potential of the proposed methodology, which makes it possible to find the potential applications for an invention applicable to distant technical fields at an early stage by using backward citations as a starting point.

### Limitations

The analysis performed in this article is subject to several limitations due to the scope of the database. First, utility model patent applications are not included in the IIP Patent database. The possibility cannot be denied that they are cited in technical fields such as 1 (Agricultural and Marine Products) and 20 (Paper), although it is thought that the influence given to the result is small since conditions are the same in the three technical fields. Second, as described in *Data*, the analysis target was limited to the six ITC classes with close numbers of IPC subclasses contained therein in order to match the conditions of technical fields.

### Future Research

As discussed above, this study omitted a couple of research issues. First, the investigation may be expanded to technology classes other than the six technology classes of the ITC and to applications filed in a year other than that of 2008. Second, a dataset may also be prepared for patent applications filed in foreign countries including Germany, China, and Korea since the analysis was conducted only for Japanese patent applications in this article. Third, there is still room to examine Originality and Generality (i.e., a concept of General Purpose Technology) defined by Trajtenberg et al. as well by using the method proposed in this article.

In *Scope of This Article*, the author mentioned that this article focuses on the technology distance only. On the other hand, it is possible to consider technical diversity as well by using three proposed technical fields consisting of same, neighboring, and distance fields. For example, in case 7 above, the author focused on only 7 forward citations classified in Distant Field, but the application has 33 forward citations, including the 7 citations above. The author believes that it will be possible to analyze the diversity of technologies as well by dividing them into three technical fields.

## Conclusion

In this article, the author studies the possibility of predictive methods using backward citations as a starting point by assessing the applicability of the insights presented by Trajtenberg et al. (1997) to Japanese applications. To verify the linkage between distant backward and distant forward citations on Japanese patent applications, the author proposes a new analysis method that makes it possible to divide a set of patent applications into three categories consisting of “same”, “neighboring”, and “distant” technical fields depending on the degree of proximity to the technical field into which the subject invention was classified. Using this new analysis method, the author creates two hypotheses and tests them for about 14,000 examined patent applications filed in 2008 with the JPO.

As a result of testing Hypothesis I, the author confirmed that Trajtenberg's insights can be applied to Japanese patent applications using citations by the examiners and IPC-based patent classifications. In other words, it was confirmed that patent applications citing backward citations categorized in a technical field distant from the invention are more likely to be cited by forward citations categorized in a technical field distant from the invention. As a result of the verification of Hypothesis Ⅱ, it was further confirmed in some technical fields that the backward citations categorized in a technical field distant from the invention are more likely to be in the same technical field as the forward citations categorized in a technical field distant from the invention.

The author believes that these results indicate the possibility of backward citations being used as a starting point for finding patent applications for inventions with potential applicability to other technical fields at an early stage.

### Notes

n1: To count forward citations received within a fixed time interval (e.g., citations received up to 5 years after a grant), it is necessary to truncate the period, which leads to a steep decline in data at the end of the period (Hall, Jaffe and Trajtenberg, 2001, pp. 25–26), which leads to a decrease in accuracy of the analysis of the data.
n2: In the United States, there is a duty to disclose to the U.S. Patent and Trademark Office any known prior art that is material to the patentability of any claim of a pending U.S. patent application. Failure to disclose relevant prior art during the prosecution of a patent application may lead to the patent being unenforceable (Code of Federal Regulations, Title 37: 37 C.F.R 1.56).
n3: Although the context is different, a similar indication was made by Thompson and Fox-Kean (2005) against Jaffe et al. (1993) which found a corresponding “control” patent issued in the same primary US patent class as the citing patent.
n4: This issue was also pointed out in Jaffe (2017), p.1366.
n5: Other than measuring technical distances based on patent classification, there are ways based on patent citation network relationships (e.g., Kay et al., 2014), or analyzing systematically the textual content of patents by natural language processing methods (e.g., Arts et al., 2018).
n6: The laid-open publication means the publication of an unexamined patent application. It is automatically published by the JPO after 1 year and 6 months from the filing date.
n7: The patent system excludes inventions lacking an inventive step from the subject from being granted even if they are new [Patent Act Article 29(2)] because granting patent rights for inventions that could easily have been made by a person having ordinary skill in the art is not only useless to the progress of technology but also prevents progress.
n8: “The Examination Guidelines for Patent and Utility Model in Japan” specifies the general procedure for determining whether or not the claimed invention involves an inventive step, so that examiners can objectively and logically make a uniform determination without variation between individual examiners (See Part 1, Chapter 2, Section 2).
n9: It is generally accepted that an invention can be grasped by dividing it into a configuration showing a solution for solving the target problem and a basic configuration that can be said to be a conventional fact (JIPA, Patent 2nd Committee, 2012).
n10: The IPC classification table is a set of classification items, and has a structure in which all technical fields are arranged in a hierarchical, tree-like structure in different levels with codes including sections, classes, and sub-classes. In this way, patent classification codes (e.g., class) indicate the technical field or fields to which the patent application relates.
n11: Similar examples are found in the case of “Insecticides/Herbicides (IPC’s Class A01N)" classified as ITC's Class 13 in combination with organic chemistry (IPC’s Class C07) as well as the case of Explosives (IPC’s Class C06) which is separated from Chemistry (IPC’s Section C) and transferred to ITC’s Class 26 (Weapons and Explosives).
n12: However, the ITC Class information has been excluded from the IIP Patent database after the data was extracted (for unknown reasons). It is necessary, therefore, to separately create an ITC table corresponding to the data of the IPC subclass in order to perform the filtering process mentioned in *Method for Extracting Data*.
n13: The JPO Examination Guidelines states that if there is prior art that the applicant finds useful, such as when making amendments, the examiner may also include the prior art information (See Guidelines, Part Ⅸ, Chapter 2, Section 2.3)They are some of the prior art that was found in the process of examining the invention. Although they do not constitute a reason for refusal, they are considered to be useful information for determining the category of Subject Applications.
n14: Although the claimed features of both inventions differ from each other, it was pointed out by the examiner that the description of the second embodiment of the present application suggests the claimed features of the forward-cited application (Official Action dated May 29, 2018).
n15: The former patent application for pulp composition (D21H) cites the present application in relation to the prior application based on Article 29–2 of the Japanese Patent Law, and thus the filing date is not significantly different from the present application.
n16: The AES is a system in which, under certain conditions, an examination is carried out earlier than usual at the request of the applicant. Considering that the target of the early examination system is green-related applications having an energy-saving effect and contributing to CO2 reduction or disaster recovery support-related applications, the potential for innovation is expected.
n17: According to the JPO Status Report 2019 (Part 2, Chapter 1, the JPO, pp. 58–59), there were 21,137 requests for accelerated examination in 2018, and the number of applications has been increasing year by year. It took 2.3 months on average to receive a first action from a request for the accelerated examination, which was significantly shorter when compared with applications not requesting the acceleration (about 24 months).

## Data Availability

The original contributions presented in the study are included in the article/Supplementary Material, further inquiries can be directed to the corresponding author.
