# Supplementary material for: Predicting the Potential Applicability to Other Technical Fields Through the Linkage Between Backward and Forward Citations
Source: Front Res Metr Anal. 2021 Oct 14;6:736687. doi: 10.3389/frma.2021.736687 (PMC8551672; doi:10.3389/frma.2021.736687)
Supplement: Supplementary file 1 [file DataSheet1.docx]

Appendix

Table S1 shows the relationship between the backward citations and the forward citations for the six selected technology classes of the ITC. Details of the backward citations are lined up in a vertical direction for each technical field in accordance with each selected technology class, whereas details of the forward citations are arranged in a horizontal direction for each technical field.

In the horizontal direction, Column X shows the details of the subject applications. Columns Y and Z show the number and the ratio of subject applications cited by forward citations categorized into the three technical fields. In the vertical direction, each row is shown separately for each selected technology class of the ITC. Each row shows the number and the ratio of subject applications citing backward citations categorized into the three technical fields. Thus, data contained in cells at the intersection of the indicated row and the indicated column shows the number and the ratio of subject applications citing backward citations and cited by forward citations.

Table S1: Table showing the relationship between the backward citations and the forward citations

for the six selected technology classes of the ITC.

Appendix

Table S2: The Results of the Matching Rate for Each Technical Field
